# Influence of acid activation on hydrogen adsorption properties of analcime-rich tuff from Turkey

**DOI:** 10.55730/1300-0527.3461

**Published:** 2022-06-02

**Authors:** Meryem AKBELEN, Nurgül ÖZBAY

**Affiliations:** 1 Department of Physics, Faculty of Science, Eskişehir Technical University, Eskişehir, Turkey; 2 Department of Chemical Engineering, Faculty of Engineering, Bilecik Seyh Edebali University, Bilecik, Turkey

**Keywords:** Analcime, hydrogen adsorption, FT-IR, TG-DTG-DTA, SEM

## Abstract

In this study, structural, thermal, and hydrogen (H_2_) adsorption properties of natural analcime-rich zeolite tuff (A) from Trakya (Turkey) and that of acid-treated forms investigated. Analcime mineral was treated with 0.1, 0.5, 1, 2, and 3 M HCl solutions at 80 °C for 5 h. Differences in structural and thermal behaviors of the analcime samples before and after acid-treatment were examined by X-ray fluorescence spectroscopy (XRF), X-ray diffraction (XRD), Fourier transform infrared spectroscopy (FT-IR), scanning electron microscopy with detector x-ray energy dispersive (SEM-EDX), thermal analysis (TG-DTG-DTA) and nitrogen adsorption methods. Micropore volume, micropore area, and specific surface area values of the acid-treated analcimes were very high compared to the natural analcime sample. It was found that the acid-treated analcime zeolites with a high SiO_2_/Al_2_O_3_ ratio exhibited higher H_2_ adsorption capacity and thermal stability. H_2_ adsorption capacities of natural and acid-treated analcimes at 77 K up to 100 kPa were found between 0.255 and 0.632 mmol/g. The hydrogen adsorption capacities of analcime samples increases in the order A < 05HA < 01HA < 1HA < 2HA < 3HA. Additionally, it was determined that the hydrogen adsorption capacity of analcime samples had an almost linear connection to the BET surface areas.

## 1. Introduction

Zeolites are porous hydrous aluminosilicates. Their 3-dimensional framework contains AlO_4_ and SiO_4_ tetrahedrons joined by common oxygen atoms. Some exchangeable cations such as Na^+^, K^+^, Ca^+2^, and Mg^+2^ are present in the channels or voids to compensate the negative charge of the network which arises from replacing Si^+4^ with Al^+3^ [1]. Analcime (NaAlSi_2_O_6_·H_2_O, ANA) is a microporous aluminosilicate zeolite that has a free opening with a main channel length of 2.6 Å. It is a sodium silicate crystal that is very common in both volcanic and sedimentary rocks. Despite being a complex structure, the crystal structure of analcime was one of the first zeolites to be determined [2]. Maximum topological symmetry of analcime, also known as leucite-type feldspathoids, is the cubic *la*3*d*. A cubic unit cell of analcime has small irregular channels that are formed of four-, six- and eight-membered distorted oxygen rings [3,4]. Average radius of the larger channels is approximately 5 Å [5]. In analcime structure, 16 positions in 24 smaller cavities hold sodium cations in the form of octahedral coordination and water molecules are located in the 16 larger channels [3, 6]. Natural analcime is a low silica zeolite with Si/Al molar ratio equal to 2 [1,7]. Its water content is directly linked to the amount of silica in its structure. As silica content increases, the number of exchangeable cations in the structure decreases [8]. Small porous zeolites (e.g., analcime) have an important place in adsorption applications.

The most common types of natural zeolites are clinoptilolite, mordenite, chabazite, and analcime. High siliceous zeolites such as mordenite, clinoptilolite, and erionite can be directly treated with strong acid solutions (e.g., HCl, H_2_SO_4_, HNO_3_) to improve the adsorption properties of natural zeolites. Some impurities or hydrated cations could block pore channel entrances and prevent access of the adsorptive molecules to micropores. The treatment of zeolites with acid solutions leads to decationization, (exchange of hydrated cations by H^+^ ions), dealumination (the removal of Al^3+^ from the structure), and sometimes degradation of the crystal structure. A partial dealumination process could increase the micropore volume and specific surface area thus making the zeolites more appropriate adsorbents for gas molecules [9–23]. Park et al. [14] found that the specific surface area and micropore volume of analcime treated with 1 N HCl solution increased from 16 to 119 m^2^/g and from 0.02 to 0.09 cm^3^/g, respectively. Cobzaru et al. [17] showed that the specific surface area and micropore volume of analcime zeolite were 14.649 m^2^/g and 0.001 cm^3^/g respectively, and they increased to 89.473 m^2^/g and 0.029 cm^3^/g for 1 N HNO_3_ acid-treated analcime.

A decline in fossil fuel sources resulting from overuse and environmental hazards led to an interest in alternative eco-friendly fuel resources such as hydrogen. Hydrogen is the simplest and most abundant element in nature; it will probably be the most significant energy source in the future if it is stored more economically and safely [24]. The kinetic diameter of a hydrogen molecule is approximately 2.89 Å. It is adsorbed through physical adsorption in porous materials. Physical adsorption occurs with van der Waals interactions under cryogenic conditions [24–26]. Zeolites have more advantages in hydrogen storage compared with metal-organic frameworks (MOFs) and nano-structured carbons thanks to better thermal and chemical stability. Certain factors including controllable cages and channels, abundancy in nature, being inexpensive, and having high mechanical and thermal stability are the main reasons for zeolites’ being preferred in hydrogen storage processes. Also, isolated and exposed cations acting as hydrogen bonding sites in these structures are one of their most remarkable features. The presence of exchangeable cations allows for the modification of characteristic properties [27]. The size and shape of the pores in nanoporous solids play a major role in hydrogen adsorption performance [28]. Zeolites are preferred as the potential adsorbent since their microporous structure is capable of adsorbing molecules at extremely low pressures.

It is known that specific surface area and the size of cations exchanged with hydrogen are effective on the hydrogen adsorption capacities of zeolites. The studies that were conducted on H-modified zeolites showed a strong relationship between adsorption at low pressures and pore size and pore volume of the zeolite. Nijkamp et al. [28] reported hydrogen adsorption of 0.7 wt% on ZSM-5 at 77 K and 0.1 MPa. They showed that there was a linear relationship between the microporous volume and the hydrogen adsorption capacity. Weitkamp et al. [29] investigated hydrogen-storage capacities of a group of zeolites (NaA, SAPO-42, sodalite, NaX, NaY, Rho, ZK-5) with different compositions and pore structures that ranged from 2.5 MPa to 10 MPa between temperatures of 293 K and 573 K. These researchers discovered that sodalite has its highest storing capacity of 0.082 wt% at 573 K and 10 MPa. H-mordenite was observed to have a better adsorption capacity (0.7 wt%) compared to HZSM5-S and H-Y up to a pressure of 0.65 bar [30, 31]. Takagi et al. [32] measured the hydrogen storage properties of two zeolites (H-YZ and H-ZSM-5) with various carbon-based materials up to 3.5 MPa at temperatures of 77 K and 303 K. They found that the hydrogen capacity of H-YZ with the surface area of 710 m^2^/g had a maximum hydrogen storage capacity of 0.6 wt% at 77 K temperature and 1 atmospheric pressure. They also stated that the amount of absorbed hydrogen by weight depends on the micropore volume of the sample. Zecchina et al. [33] explored hydrogen adsorption for a group of chabazite zeolites (H-SSZ-13, H-ZSM-5, and H-SAPO-34), and discovered that high silica zeolite H-SSZ-13 (Si/Al = 11.6), which has a chabazite structure, was the most effective zeolite for hydrogen storage with a maximum storage capacity of 1.28 wt% at 77 K and 0.92 bar. Regli et al. [34] examined hydrogen adsorption properties of the chabazite materials of H-SSZ-13 (Si/Al = 11.6), H-SAPO-34, and H-CHA (standard chabazite zeolite, Si/Al = 2.1), volumetrically at 77 K temperature. They found that H-SSZ-13 showed the best behavior in terms of hydrogen adsorption. Torres et al. [35] studied H_2_ adsorption on acid-treated chabazite zeolites by theoretical analyses. Langmi et al. [36] reported that NaY zeolite showed the highest hydrogen adsorption capacity of 1.81 wt% (at 77 K and 15 bar). They found that the hydrogen adsorption capacities of A, X, Y, and RHO zeolites are directly related to their surface area. Jhung et al. [37] observed that certain zeolites (e.g., faujasite, mordenites and ZSM-5) that contain aluminum in various proportions had hydrogen adsorption at low temperatures that were directly proportional to the increase of zeolite-aluminum components. They also discovered that adsorption capacity of zeolites with similar aluminum ratios increased in the order mordenite > ZSM-5 > faujasite due to different pore sizes. Kyong-Hwan [38] investigated the effect of the framework structure, surface area, and pore volume of the zeolites on hydrogen storage on MOR, FAU, and MFI type microporous zeolites at high pressure (<100 bar). The largest hydrogen storage was obtained on the ultra-stable Y (USY) zeolite (0.4 wt%). In addition, this study showed that hydrogen storage capacity depends mostly on the pore volume of the zeolite. Erdoğan-Alver and Sakızcı [39] investigated hydrogen adsorption properties for acid forms of clinoptilolite (CLN), mordenite (MOR) and chabazite (CHA) zeolites. They reported the hydrogen storage capacities of 0.308 wt%, 0.474 wt%, and 0.482 wt%, obtained at 77 K and 100kPa, for H-MOR, H-CHA, and H-CLN respectively.

The mineral deposits with analcime-rich zeolite tuffs are available within a large area in Turkey. However, there are very few studies of the hydrogen adsorption on acid forms of analcime in Turkey. The novelty of this study was to investigate the effect of acid on the structure and the thermal and hydrogen adsorption properties of analcime-rich zeolite tuffs that were found in the Trakya region of Turkey.

## 2. Experimental

### 2.1. Materials and methods

In this study, zeolite, which is rich in the natural analcime, was used as obtained from the Trakya region of Turkey. First, zeolite rock samples were milled and sieved to obtain particle dimensions that are smaller than 45 μm. Acid-treated forms of analcime samples were prepared by adding 5 gr of zeolite to 100 mL hydrochloric acid solution at different concentrations (0.1 M, 0.5 M, 1 M, 2 M, and 3 M) at 80 °C for 5 h. A back-cooled magnetic stirrer and hot plate system were used during modified processes of zeolite samples. Following acid treatment, the samples were filtered and washed several times with deionized water to remove acid residues. Then, all of the acid forms were dried at room temperature and kept in an oven at 105 °C for 20 h prior to experiments. Then, all samples were stored in a desiccator. The natural sample was labeled as A and the acid-treated samples were labeled as 01HA, 05HA, 1HA, 2HA, and 3HA (the numbers refer to different acid concentrations). HCl was obtained from Merck (Darmstadt, Germany), and all solutions were prepared with deionized water.

### 2.2. Instrumentation

The chemical composition of natural and acid-treated analcime samples was obtained from the powder samples that were fused with lithium tetraborate using an XRF device, Rigaku ZSX Primus. X-ray diffraction (XRD) analysis was performed with a D8 Advance Bruker device using CuKα radiation (λ = 1.54 Å) at 40 kV and 40mA between 4° and 40° (2θ).

FT-IR spectra of the analcime samples were recorded using a Perkin Elmer Spectrum 100 model spectrophotometer in a wave number range of 450–4000 cm^−1^ with the potassium bromide (KBr) pellet technique.

The morphologies and elemental compositions of analcime samples were analyzed by scanning electron microscopy (SEM) using a ZEISS ULTRAPLUS instrument equipped for energy-dispersive x-ray (EDX) spectroscopy. Analcime samples were coated with gold-palladium at 50 mA for 1 min to improve conductivity during an investigation by SEM.

TG-DTG-DTA experiments of the natural and acid-treated analcime samples were carried out by a simultaneous Setsys Evolution Setaram thermal analyzer between 30 and 1000 °C in the aluminum crucibles, using ~30 mg of a sample at a linear heating rate of 10 °C min^−1^. Empty alumina crucibles (100 μL) were used as the reference.

Nitrogen adsorption and desorption isotherms were measured by a Micromeritics 3Flex device at a liquid nitrogen temperature of 77 K to determine the textural properties of natural and acid-treated analcime samples. High-purity (99.99%) nitrogen gas was used in N_2_ adsorption/desorption experiments. Specific surface areas were calculated from the adsorption branch using the Brunauer–Emmett–Teller (BET) method under a vacuum environment at 300 °C following 12 h of degassing. Total pore volume was estimated by measuring the volume of the maximum nitrogen gas that was adsorbed at a P/P_o_ = 0.98 partial pressure. The pore size distribution and average pore diameter were calculated based on the Barrett-Joyner-Halenda (BJH) model. The *t*-plot method was applied to measure micropore size using the de Boer equation.

H_2_ adsorption experiments of the natural and acid-treated analcime samples were carried out using the Autosorb 1-C volumetric equipment (Quantachrome Instruments) at 77 K up to 100 kPa pressure. High-purity (more than 99% purity) hydrogen gas was used. All analcime samples underwent a degassing process at 300 °C for 12 h prior to hydrogen adsorption experiments.

## 3. Results and discussion

### 3.1. Chemical analysis

The chemical compositions of natural and acid-treated analcimes, expressed in terms of oxide species, are presented as % mass in Table 1. There were changes in the chemical composition of natural analcime during acid treatment. Although Na, Ca, and Fe cations could vary majorly as the acid concentration rises, Mg and K cations could move away partially only since they had a stronger bond with the analcime. The SiO_2_/Al_2_O_3_ ratio increased from 4.62 to 6.15 as a result of acid treatment [14, 17, 19, 40].

### 3.2. X-ray analysis

**Figure 1:**
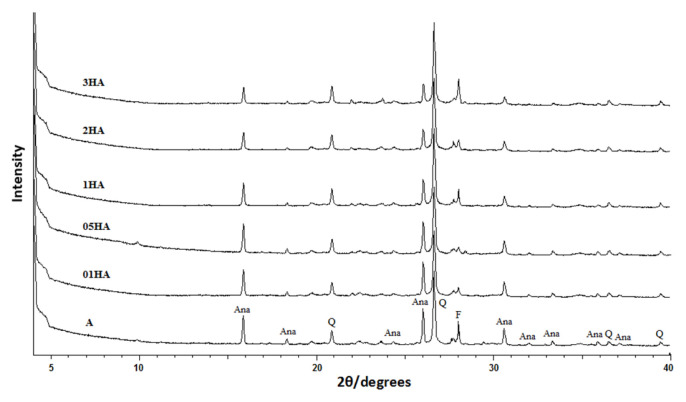
XRD patterns of the natural and acid-treated analcimes (Ana: analcime; Q: quartz; F: feldspar).

X-ray diffraction (XRD) patterns of natural analcime (A) and acid-treated analcime samples (01HA, 05HA, 1HA, 2HA, and 3HA) are presented in Figure 1. XRD patterns were mainly formed by the analcime (d = 5.58, 4.83, 3.41, 2.91, and 2.49 Å) along with natural zeolite quartz (4.25, 3.34, 2.45, and 2.28 Å) and feldspars (d = 3.21 Å) [41]. While the study observed a gradual reduction (d= 5.58, 3.41, and 2.91 Å) in the characteristic analcime peak intensities of the H forms after the acid treatment, there was an increase in quartz peak intensities (d = 4.25 Å and d = 3.34 Å) (Figure 1) [14, 17, 19]. This probably stems from the fact that the acid treatment dealuminated the analcime structure and partially destroyed it. The impurities in the structure (e.g., feldspar, quartz) did not resolve and remained in the structure with the acid process that was applied on analcimes [13]. Changes in the intensity of the feldspar peak of some analcime samples can be attributed to the migration of cations and the removal of water molecules from the structure due to heating.

### 3.3. FT-IR analysis

**Figure 2:**
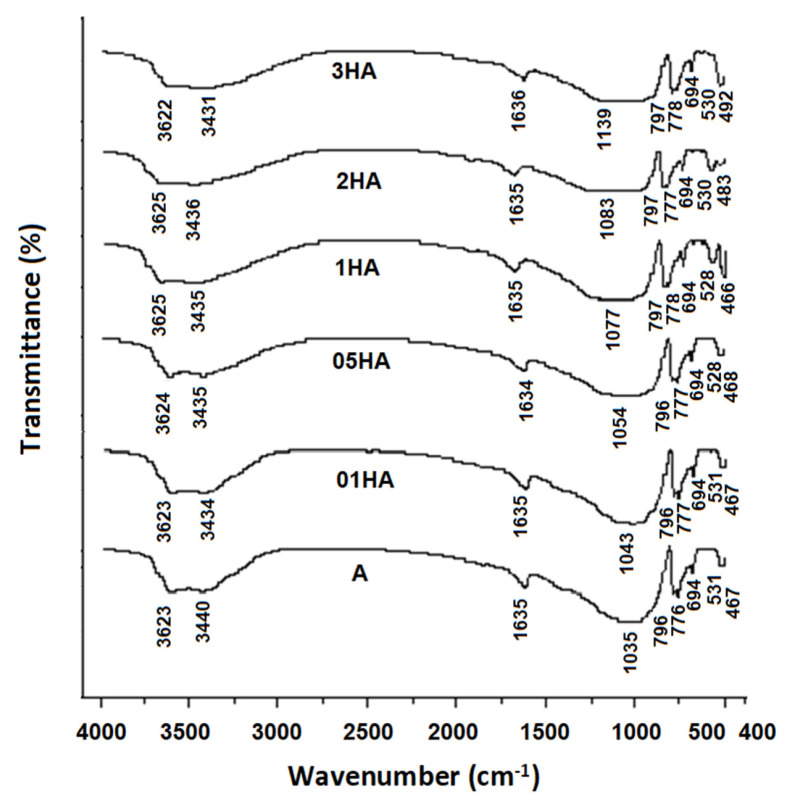
FT-IR spectra of natural and acid-treated analcime samples.

FT-IR results of natural and acid-treated analcime samples are given in Figure 2. In FT-IR spectrum of analcime samples, the bands at 1600–3700 cm^−1^ indicated the presence of zeolite water [42]. The bands at 3622–3625 cm^−1^ and 3431–3440 cm^−1^ were related with the asymmetric and symmetric stretching vibrations of hydroxyl groups, respectively [43]. The band at about 1634–1636 cm^−1^ was attributed to the fundamental vibration of bending hydroxyl groups [44,45].

The absorption bands in the range of 400–1300 cm^−1^ correspond to characteristic bands of aluminosilicate minerals such as zeolites. The strongest asymmetric stretching vibration band shifted to higher wavenumber values (1035–1139 cm^−1^) with the loss of aluminum cations in the framework tetrahedral sites [42,46]. This is due to the dealumination from the structure of analcime zeolite as shown from XRF (Table 1). The bands at 796–797 cm^−1^ and 776–778 cm^−1^ were assigned to the symmetric stretching vibration of external tetrahedral T-O (T=Si, Al) bonds. The band observed at 694 cm^−1^ was attributed to the symmetric stretching vibration of internal tetrahedral T-O bonds. The band at 528–531 cm^−1^ could be assigned to potassium feldspar, which was consistent with the XRD patterns of analcime samples [47]. The band appearing at 466–468 cm^−1^ was associated with the T-O bending vibration of the SiO_4_ and AlO_4_ [7, 42, 48].

### 3.4. SEM/EDX analysis

**Figure 3:**
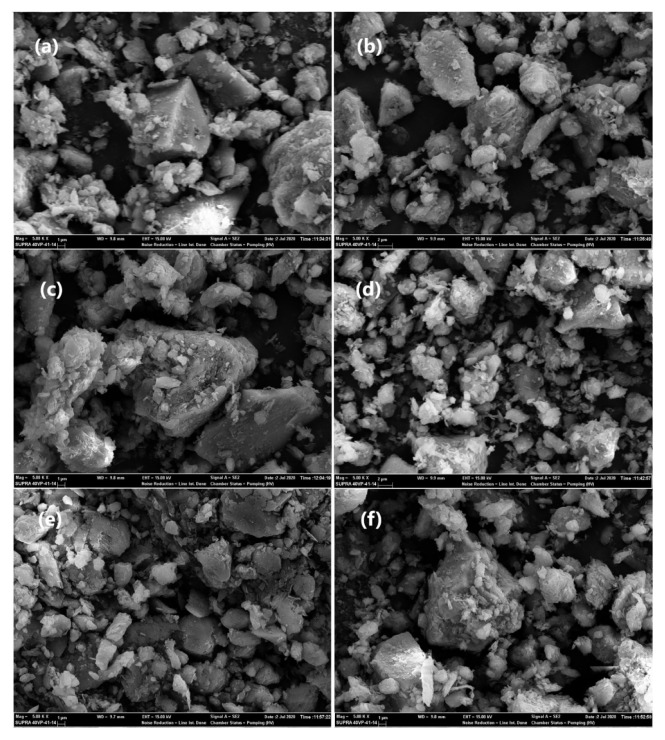
SEM images of (a) natural, (b) 01HA, (c) 05HA, (d) 1HA, (e) 2HA, and (f) 3HA samples.

SEM images of natural and acid-treated analcime samples are given in Figure 3. As reported by the previous literature [1,40,49–53], sedimentary analcime minerals are commonly present as euhedral crystals in the form of trapezohedron and cube-octahedral crystals. In Figure 3, the analcime sample appears as small euhedral analcime crystals associated with quartz, as shown in Figures 3a–3f. It can be seen from SEM images that the acid treatment did not have a significant influence on the morphology of analcime samples.

The elemental compositions of the analcime sample and its cationic forms were determined using EDX performed with 2 different random points. The presence of analcime was verified by XRD and SEM-EDX analyses. EDX analysis demonstrated the presence of Al, and Si, Na, K, Mg, and Ca elements, which constitute the analcime composition (Table 2). According to EDX results, Na content of the analcime sample was greatly reduced after acid treatment [54].

### 3.5. Thermal properties

Dehydration of the analcime zeolites is a simple process since water molecules in the crystal structure of the analcime are clearly present only in one position [5]. Thermal properties of analcime minerals were studied by several researchers [54–56] and no phase transformation at high temperatures during the thermal analysis of the cubic analcime was observed [54].

**Figure 4:**
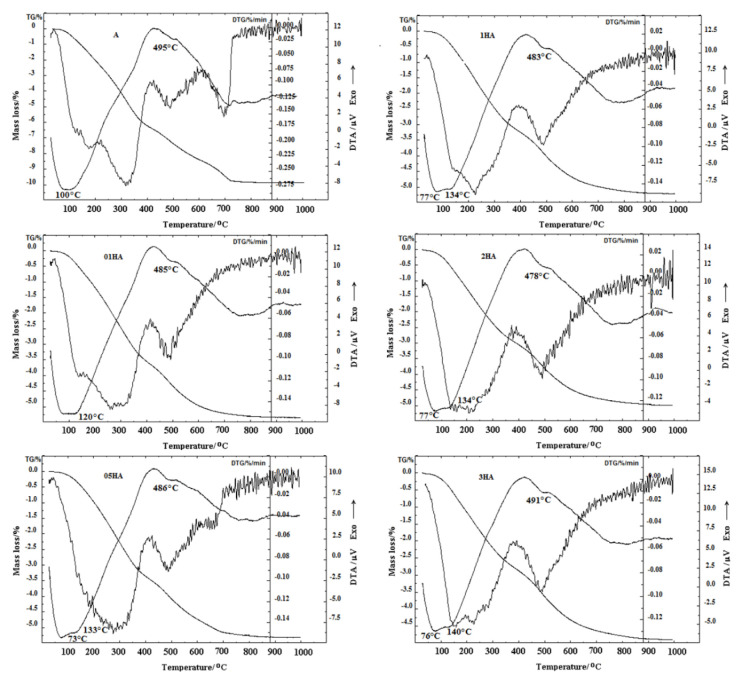
TG-DTG and DTA curves for the natural and acid-treated analcime samples.

The TG–DTG–DTA curves of the natural and acid-treated analcimes are shown in Figure 4. The thermal analysis findings were consistent with previous research results. The DTA curves that were obtained for the zeolite samples were fundamentally similar. The natural analcime (A) and 01HA samples showed two endothermic peaks at 100–120 at 485–495 °C, which are different from the other samples. The samples 05HA, 1HA, 2HA, and 3HA showed three endothermic peaks of 73–77, 133–140 at 478–491 °C. The TGA-DTG curves of the analcime samples showed that thermal events occur in three temperature intervals: 30–400 °C, 400–750 **°C**, and 750–1000 **°C**. The large and rapid mass loss (0.98 %–2.06 %) that was observed in the temperature range between 30 and 200 **°C** was caused by the desorption of physically adsorbed water and loosely bound water (Table 3). The mass loss of analcime samples was high at temperatures of 200 to 400 **°C** and was ranging from 1.70% to 4.17%. Mass losses of the analcime samples at temperatures of 400 to 750 **°C** were around 1.71% to 3.55%. The main reason for mass loss between 200 **°C** and 750 **°C** was the removal of hydroxyl groups from the analcime structure. Smaller mass losses (0.08%–0.17%) between 750 **°C** and 1000 **°C** resulted from the removal of water that was associated with silanol nets.

Partial dealumination improves the thermal stability of the analcime samples and increases their hydrophobicity [12,13]. As a result, the H-forms of analcime had both less mass loss and lower endothermic peak temperature compared to the natural sample (Figure 4 and Table 3) [57]. The total mass losses of A, 01H, 05HA, 1HA, 2HA, and 3HA were determined to be 9.88%, 5.34%, 5.31%, 5.21%, 4.99%, and 4.89%, respectively, at 1000 **°C**.

### 3.6. Specific surface area and porosity

**Figure 5:**
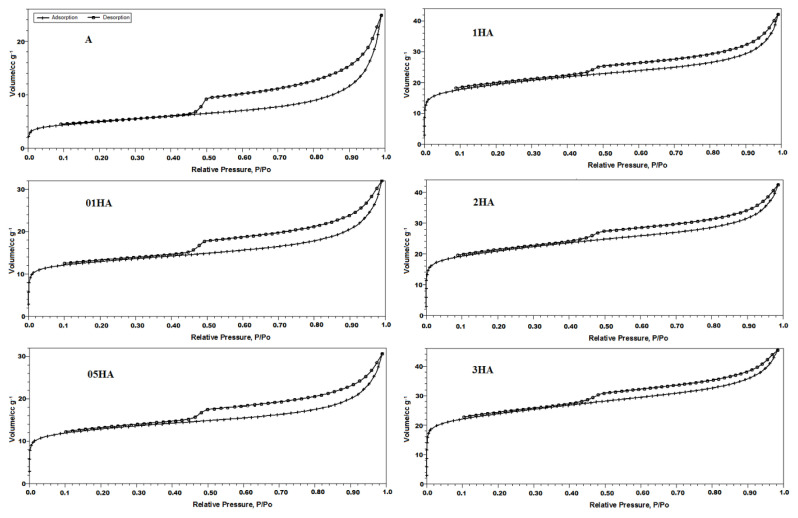
Adsorption-desorption isotherms of N_2_ at 77 K for natural and acid-treated analcimes.

**Figure 6:**
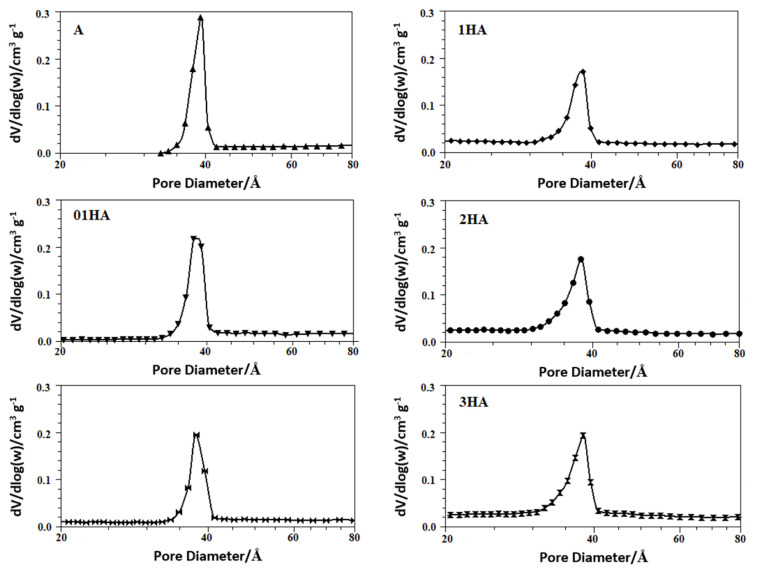
BJH desorption pore-size distributions of natural, 01HA, 05HA, 1HA, 2HA, and 3HA samples.

The isotherms of N_2_ adsorption/desorption for the natural and acid-treated analcime samples and their pore size distributions are presented in Figures 5 and 6; their textural characteristics are listed in Table 4. All analcime samples showed a Type-IV isotherm, characteristic of mesoporous and microporous solids [58]. Type H3 hysteresis curves were observed, proving the presence of slit-shape pores in analcime zeolites [59]. The hysteresis curves in the multilayer range of isotherms are related to the capillary condensation that occurs in mesoporous. The study observed that the adsorbed amount increased quite sharply in the isotherm curve at low pressures (P/P_o_ ~ 0.4) related to the presence of mesopores as well as micropores. Surface areas were calculated using both the Brunauer–Emmett–Teller (BET) equation model and the Langmuir equation model (Table 4). The pore size distributions were determined from the desorption branch using the BJH adsorption method. In this pore diameter curves of these samples, a sharp peak was observed at 40 Å (Figure 6).

The pore entrance of the small eight-membered rings in the natural analcime structure prohibits the adsorption of large nitrogen molecules (3.7 Å) [60]. In the present study, acid-treated analcime zeolites demonstrated remarkable nitrogen adsorption due to dealumination and decationation as well as the dissolution of any amorphous materials that blocked the pore (Figure 5) [14, 17, 19]. New micropores were formed by removing aluminum cations by leaching from the framework position of the acid-treated zeolites in the structure. Furthermore, cations, which occupy micropore channels, caused clogging before the acid treatment and prevented gas molecules from entering the structure. These hydrated cations are exchanged with H^+^ after the acid treatment and reduce the blocking effects caused by the cations in micropore channels [9,10,14,17,61]. Thus, the micropore volume and specific surface area values increased significantly due to the reduction of the average pore diameters of acid-treated analcimes (Table 4) [13,57]. The specific surface area of the zeolites in the adsorption branches ranged from 17.41 to 86.64 m^2^/g in the linear part of the BET (Brunauer-Emmet-Teller) curves [62]. The acid treatment caused the specific surface area of the natural analcime to be nearly five-fold increase (Table 4). Although the specific surface area and total pore volume of the natural analcime were 17.41 m^2^/g and 0.036 cm^3^/g, respectively, they increased to 86.64 m^2^/g and 0.069 cm^3^/g, respectively, after 3 M HCl solution treatment. An increase in the HCl concentration causes an increase in the specific surface area, assisting micropore formation [15,21,59]. The surface areas for samples 01HA and 05HA were calculated as approximately ~47 m^2^/g. This result could be attributed to the blocking of the pore channels of the sample with some large cations or some dissolved impurities during the modification of analcime with 0.5 M HCl acid solution [9].

### 3.7. Adsorption of H_2_

**Figure 7:**
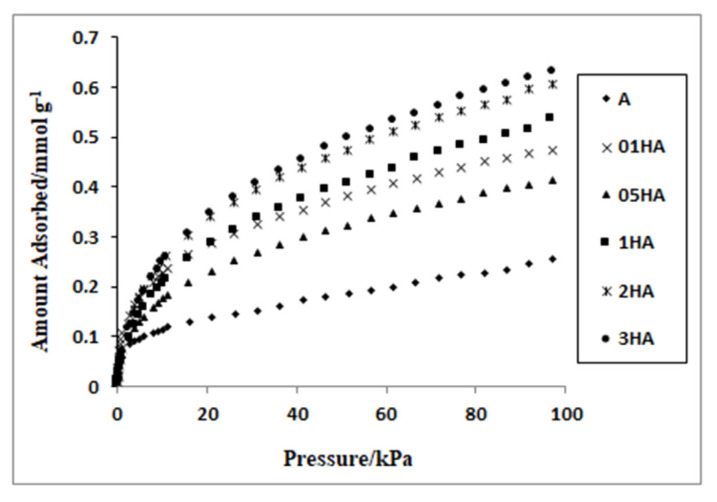
H_2_ adsorption isotherms of natural and acid-treated analcimes at 77 K.

The hydrogen (H_2_) adsorption isotherms of natural and acid-treated analcime samples up to 100 kPa at 77 K are shown in Figure 7. Theoretically, the natural form of analcime has enough pore openness for the diffusion of small molecules such as H_2_ (2.89 Å). However, cation size in analcime structure, its position, and the possible blockage effects of impurities in the main channels prevent the diffusion of this molecule. As seen in the present study, the effectiveness of an adsorbent for adsorbed gas molecules depends on surface areas (Table 4). The acid treatment has a positive effect on the hydrogen adsorption capacity since it increases the microporous volume and surface area of the analcime [38,39].

The acid treatment causes the replacement of exchangeable cations with small size H^+^ ions and the removal of some aluminum ions from the framework. Acid treatment also leads to the dissolution of some impurities inside channels within the structure and production of new pores. As a result of this activation process of the analcime, specific surface area, gas sorption capacity, SiO_2_/Al_2_O_3_ ratio, and surface acidity can vary significantly. A change in the SiO_2_/Al_2_O_3_ ratio of the natural zeolites considerably affects the adsorption properties of these samples. In addition, since the adsorption of impurities is usually very low, the adsorption capacity of natural zeolites can be improved by the dissolution of these nonzeolite materials [9,10,13,14,17,19,61].

The adsorbed absolute amounts for whole analcime samples per gram are summarized in Table 4. The hydrogen (H_2_) adsorption capacities of natural and acid-treated analcimes were found as 0.255–0.632 mmol/g at 77K up to 100 kPa pressures. The hydrogen adsorption capacities of analcime samples increases in the order A < 05HA < 01HA < 1HA < 2HA < 3HA. The hydrogen storage capacities of the natural analcime and that of acid-treated forms were almost linear in relation to the BET surface areas. The large BET surface values of zeolites were found to be related to more gas molecules reaching the spaces inside the pores. Natural analcime zeolite that was treated with 3 M hydrochloric acid solution had the highest hydrogen adsorption capacity with a surface area of 86.64 m^2^/g (Table 4). The hydrogen adsorption capacity of the natural analcime sample (A) increased from 0.255 mmol/g to 0.632 mmol/g. It was observed that the 05HA zeolite had the lowest hydrogen adsorption capacity (0.413 mmol/g) among the acid-treated analcime samples. The reason that 05HA had less adsorption capacity than 01HA may be attributed to the high possibility of the blocking of the pore channels’ entrance by some cations and impurities.

## 4. Conclusions

Natural and acid-treated analcime samples were characterized using XRF, XRD, FT-IR, SEM-EDX, TG-DTG-DTA, and nitrogen adsorption methods. It was seen that the chemical composition and thermal behavior of analcime samples were affected by the acid treatment. The acid treatment of natural analcime with HCl leads to an increase of thermal stability and hydrophobicity. TG analysis of all analcime samples showed that the mass loss of the acid-treated samples was lower than the natural form. Partial dealumination of analcime resulted in a significant increase in microporosity. The micropore volume, micropore area, and specific surface area of the acid-treated samples were remarkably high compared with the natural analcime samples.

The adsorption activity of analcime zeolite was improved by removing the blockage in the pore channels of the analcime structure via decationation and dealumination with HCl acid. A maximum hydrogen storage capacity of 0.632 mmol/g (0.127 wt%) was observed for 3HA sample. Consequently, an increase in the Si/Al ratio with an acid treatment made this zeolite interesting for its hydrogen adsorption.

## Figures and Tables

**Table 1:** Chemical compositions of natural and acid treated analcime samples determined using XRF.

Chemical Analysis (%)	A	01HA	05HA	1HA	2HA	3HA
SiO_2_	66.25	68.93	70.47	71.38	72.85	73.76
Al_2_O_3_	14.32	13.59	13.50	12.96	12.11	11.99
Fe_2_O_3_	2.54	2.50	1.92	1.80	1.57	1.56
MgO	0.73	0.60	0.56	0.55	0.55	0.52
CaO	2.38	1.05	0.87	0.61	0.56	0.54
K_2_O	1.90	1.89	1.86	1.85	1.84	1.81
Na_2_O	3.54	3.52	3.32	2.77	2.57	2.39
TiO_2_	0.34	0.32	–	0.32	0.31	0.26
P_2_O_5_	0.14	–	–	–	–	–
LOI	7.86	7.60	7.50	7.76	7.64	7.17
SiO_2_/Al_2_O_3_	4.60	5.07	5.22	5.50	6.01	6.15

**Table 2:** Average elemental compositions of natural and modified analcime samples.

Element,%	A	01HA	05HA	1HA	2HA	3HA
O	57.08	58.90	60.21	61.93	62.05	62.82
Si	26.32	27.41	29.22	29.48	31.09	32.95
Al	9.35	8.73	7.43	6.52	5.30	2.91
Fe	1.28	0.99	0.44	0.44	0.43	0.37
Mg	0.44	0.43	0.38	0.31	0.27	0.15
Ca	1.22	0.86	0.20	–	–	–
K	0.94	0.76	0.73	0.72	0.73	0.72
Na	3.27	1.92	1.39	0.60	0.13	0.08
Ti	0.05	–	–	–	–	–
P	0.05	–	–	–	–	–

**Table 3:** Mass loss (%) of natural and acid-treated analcime samples at different temperature ranges.

Sample	30–200 °C	200–400 °C	400–750 °C	750–1000 °C	Total mass loss (%)
A	2.06	4.17	3.55	0.10	9.88
01HA	1.18	2.37	1.71	0.08	5.34
05HA	0.98	2.38	1.85	0.10	5.31
1HA	1.24	2.01	1.81	0.15	5.21
2HA	1.27	1.78	1.79	0.15	4.99
3HA	1.17	1.70	1.85	0.17	4.89

**Table 4:** N_2_ and H_2_ adsorption data of natural and acid treated analcime samples.

Sample	H_2_ (mmol/g) 77 K	Surface area BET (m^2^/g)	Surface area Langmuir (m^2^/g)	Micropore area t-plot (m^2^/g)	Micropore volume t-plot (cm^3^/g)	Cumulative pore volume BJH (cm^3^/g)	Total pore volume (cm^3^/g)	Average pore diameter BJH (Å)
A	0.255	17.41	18.94	4.220	0.002	0.037	0.036	90.49
01HA	0.474	47.05	58.33	30.061	0.012	0.042	0.047	78.46
05HA	0.413	46.84	94.84	28.458	0.011	0.034	0.045	69.76
1HA	0.536	70.10	80.93	36.487	0.015	0.045	0.062	63.71
2HA	0.607	75.76	88.49	41.295	0.017	0.047	0.064	57.86
3HA	0.632	86.64	102.45	48.983	0.020	0.047	0.069	54.59
